# Adapting for endocytosis: roles for endocytic sorting adaptors in directing neural development

**DOI:** 10.3389/fncel.2015.00119

**Published:** 2015-04-08

**Authors:** Chan Choo Yap, Bettina Winckler

**Affiliations:** Department of Neuroscience, University of VirginiaCharlottesville, VA, USA

**Keywords:** clathrin, signaling endosomes, TrkB, numb, disabled, doublecortin, migration

## Abstract

Proper cortical development depends on the orchestrated actions of a multitude of guidance receptors and adhesion molecules and their downstream signaling. The levels of these receptors on the surface and their precise locations can greatly affect guidance outcomes. Trafficking of receptors to a particular surface locale and removal by endocytosis thus feed crucially into the final guidance outcomes. In addition, endocytosis of receptors can affect downstream signaling (both quantitatively and qualitatively) and regulated endocytosis of guidance receptors is thus an important component of ensuring proper neural development. We will discuss the cell biology of regulated endocytosis and the impact on neural development. We focus our discussion on endocytic accessory proteins (EAPs) (such as numb and disabled) and how they regulate endocytosis and subsequent post-endocytic trafficking of their cognate receptors (such as Notch, TrkB, β-APP, VLDLR, and ApoER2).

## Introduction

The development of the mammalian brain involves a multitude of precisely coordinated processes, including proliferation and differentiation of neural stem cells followed by migration of newborn neurons from their birthplace to their final destination, elaboration of axons and dendrites, and finally synapse formation (LoTurco and Bai, 2006; Kawauchi and Hoshino, 2008; Liu, 2011; Lewis et al., 2013; Wu et al., 2014). During cortical development, neurons born in the ventricular zone (VZ) migrate radially by moving along radial glia fibers to form proper cortical layers. Interneurons also migrate long distances, but follow a tangential migratory route that does not involve migration along radial glia processes (Guo and Anton, 2014). Defective neuronal migration causes neurodevelopmental disorders such as mental retardation, Lissencephaly, epilepsy, and others (Jamuar and Walsh, 2014). Other cell types in the brain also migrate to reach their final distribution, including oligodendrocyte precursor cells (Choe et al., 2014) and microglia (Arnò et al., 2014), but less is known about the cues and receptors that guide their movements.

Directional migration in any cell type requires cell polarization and regulated cycles of adhesion and de-adhesion (Vicente-Manzanares and Horwitz, 2011). Multiple extracellular ligands and their receptors mediate the elaboration of polarized cell morphology and of directional protrusions by dynamically regulating linkages to cytoskeletal elements. Cadherins are often implicated in cell-cell contact mediated guidance (such as might occur for radial migration along radial glial fibers) whereas integrins are well known to mediate migratory behavior for cell-extracellular matrix interactions (such as migrating along basement membranes). In addition, polarized membrane addition and removal contributes to directional motility (Vitriol and Zheng, 2012). Because migratory behavior is mediated by membrane receptors, their intracellular trafficking is essential for regulating the responsiveness of migratory cells to extracellular guidance cues. This is also true for neuronal migration. Precise sorting of membrane guidance receptors to specific regions in migratory neurons, regulating surface levels via endocytic trafficking of the receptors, as well as decisions to recycle or degrade receptors post-endocytically are crucial for brain development, but most of the detailed mechanisms are still poorly understood. Since ligand-receptor systems continue to play important roles past the migratory stage for axon and dendrite growth and for synaptogenesis, understanding membrane traffic in brain development will shed light on many normal brain processes as well as on dysfunctions of the nervous system (Yap and Winckler, 2012).

Not surprisingly, multiple genes important for proper brain development have molecular roles in endocytic membrane traffic. One prominent example is numb, an evolutionary conserved protein originally identified as a cell fate determinant during peripheral and CNS development in *Drosophila* (Uemura et al., 1989). The molecular role of Numb is as an endocytic co-adaptor that interacts with cargos and with endocytic machinery, such as the clathrin adaptor AP-2 and the endocytic accessory proteins Eps15. It thus associates with the clathrin complex for clathrin-dependent endocytosis of membrane proteins (Salcini et al., 1997; Santolini et al., 2000; Smith et al., 2004; McGill et al., 2009). How exactly is neural development regulated by an endocytic adaptor, such as numb? The mechanisms involve regulating the endocytosis, trafficking, and signaling of developmentally relevant receptors. Which other endocytic adaptors play roles in receptor endocytosis during neural development? Which receptors require which co-adaptor (numb or another co-adaptor) for endocytosis and how is their endocytosis important for normal development? Are endocytic co-adaptors also involved in post-endocytic trafficking of the receptor? These are some of the question we will explore in this review. In particular, we will discuss co-adaptor proteins that are involved in clathrin-mediated pathways via AP-2, such as Numb, Dab family proteins, and DCX, and explore their respective roles in brain development.

## Clathrin-Mediated Endocytosis: The Basic Process

Many excellent reviews have been published about clathrin-mediated endocytosis, and we only want to briefly summarize the most important facts here. Most endocytosis of receptors in mammalian cells is mediated by assembling a multimeric complex between membrane receptors (i.e., cargos), adaptor protein complexes (AP-2, in particular) and a coat lattice composed of many trimers (“triskelions”) of clathrin (McMahon, 1999; McMahon and Boucrot, 2011; Merrifield and Kaksonen, 2014). Due to better and better imaging, the sequence of events in assembling this multimeric complex at the plasma membrane (called a “clathrin-coated pit”, or CCP) is becoming better understood on a mechanistic level: A nucleation complex (including FCHO proteins, Eps15, and intersectins) associates with the plasma membrane via PIP(4, 5)P_2_ binding and initiates CCP formation. The tetrameric AP-2 adaptor is recruited to the nucleation sites and serves as a major hub for recruiting other accessory proteins as well as cargos to the forming CCP. As clathrin coat assembly progresses, the membrane is increasingly deformed into a larger clathrin-coated invagination that accumulates cargos. Ultimately, the invaginating membrane cup fissions to bud a clathrin-coated vesicle into the cell interior. The clathrin coat is rapidly removed by uncoating enzymes, and the endocytic vesicle delivers its cargos into the endosomal system by fusion with early endosomes. In addition to the essential core components of cargo, AP-2 adaptor, and clathrin, many other endocytic accessory proteins (EAPs) or “co-adaptors” associate with CCPs and aid in cargo selection, in the efficiency of cargo enrichment at the CCP, and in execution of subsequent membrane deformation, fission, uncoating, and endosomal fusion events. In addition to clathrin-mediated endocytosis, there are non-clathrin endocytosis pathways that are less well understood. It was found recently that in a mammalian cell line 95% of cargos enter the cells via clathrin-mediated endocytosis (Bitsikas et al., 2014). Surprisingly, this includes GPI-linked proteins, which had been thought to enter primarily via non-clathrin pathways. If this overwhelming preponderance of clathrin- vs. non-clathrin pathways also holds in the nervous system remains to be seen.

### Tetrameric Adaptor Complexes

To date, five tetrameric adaptor complexes (AP-1 through AP-5) have been identified in mammals. Each adaptor complex consists of two large (α, γ, δ, ε, ζ and β1–5), one medium (μ1–5), and one small subunit (σ1–5) (Fölsch, 2008; Hirst et al., 2011). The large subunits mediate binding to the target membrane (via specific phosphoinsoitide lipids), to clathrin, and to other accessory proteins (Brodsky et al., 2001). The medium subunit (μ) directly interacts with the “tyrosine-based” sorting signals (YXXΦ) in the cytoplasmic tail of cargo molecules [“X” means any amino acid can be at that position whereas “Φ” means an amino acid with a bulky hydrophobic side chain can be at that position.]. The small σ subunits are involved in the recognition of “dileucine-based” sorting motifs ([DE]XXXL[LI]) [square brackets mean that either of the bracketed amino acids can be at that position], such as the one found in the cation-independent mannose 6-phosphate receptor (CI-M6PR). AP-1 and AP-2 are both components of clathrin-coated vesicles, either for sorting at the trans-Golgi network (TGN)/endosomes (AP-1) or for endocytosis from the plasma membrane (AP-2). Both AP-3 and AP-4 are associated with TGN/endosomal membranes, and either mediate endosome/lysosome delivery (AP3 and AP4) or the biogenesis of specialized secretory vesicles in neurons (AP3) from the TGN (Faúndez et al., 1998; Mullins and Bonifacino, 2001; Robinson and Bonifacino, 2001). AP-3 also binds clathrin, whereas AP-4 is not significantly enriched in clathrin-coated vesicles and has no clathrin box consensus motif in its β4 subunit, suggesting it acts independently of clathrin (Simpson et al., 1996; Dell’Angelica et al., 1997, 1998; Hirst and Robinson, 1998; Hirst et al., 1999; Barois and Bakke, 2005). AP-5 was only recently identified and shown to associate with late endosomal/lysosomal compartments and might regulate the sorting from early endosome to lysosomes (Hirst et al., 2011). Importantly, mutations in adaptor protein complexes have been implicated in several neurodevelopmental disorders, such as Mednik syndrome (which manifests with abnormal Copper metabolism due to a copper transporter defect) and hereditary spastic paraplegia (HSP; Hirst et al., 2013).

#### Clathrin-Mediated Endocytosis Via AP-2: Constitutive or Regulated?

Much of the regulation of endocytosis likely involves regulating when and where to form a coated pit and which cargo proteins to recruit into it. Developmental processes that rely on endocytosis of specific receptors are thus subject to regulation of cargo/adaptor/coat interactions. Some of these regulatory mechanisms have been worked out, and many others will certainly be discovered in the future (Traub and Bonifacino, 2013). For example, AP-2 recruitment to the plasma membrane requires PI(4, 5)P_2_ clustering, and can be inhibited by phosphorylation of μ2 by the kinase AAK (Conner and Schmid, 2002; Ricotta et al., 2002). Upon binding to PI(4, 5)P_2_, AP-2 undergoes a large conformational change that exposes the binding sites on the μ2 subunit for tyrosine-based endocytic signals on cargo receptors. Phosphorylation of tyrosine-based signals on the cargos similarly inhibits their binding to AP-2. Endocytosis of receptors is thus regulated from two ends: regulating receptor affinity for endocytic adaptors, and regulating affinity of the adaptor for the sorting signal on the receptor. Endocytosis of receptors is thus far from constitutive, but rather highly regulated.

#### AP-2 Function in Neurons

AP-2 has been implicated in clathrin-mediated endocytosis of membrane proteins important for synaptic plasticity and neurotransmission (Kononenko et al., 2014). In addition, clathrin-mediated endocytosis is needed during development for axon and dendrite outgrowth and for pathfinding. Clathrin/AP-2 mediated endocytosis has been associated to play an important role in adhesion disassembly during cell migration. For example, clathrin coated pits are enriched at adhesive contacts with matrix substrates and co-localized with adhesion receptors in migratory neurons. Inhibition of dynamin or clathrin function impaired neuronal migration both *in vivo* and *in vitro*, and coincided with a shift in the distribution of adhesion proteins from the original region proximal to the cell body to the rear of the cells (Shieh et al., 2011). In addition, AP-2 also interacts with the tyrosine-based motif of L1-CAM to mediate its endocytosis at axonal growth cones, a process crucial for its local recycling and detachment from adhesive environment to promote axon elongation and outgrowth (Kamiguchi et al., 1998). Additionally, endocytosis of L1-CAM in dendrites is also essential for targeting L1-CAM to axons via an indirect transcytosis pathway (Wisco et al., 2003; Yap et al., 2008).

### Monomeric Endocytic Co-Adaptors

Different endocytic cargos have different endocytic signals and can be endocytosed via different molecular assemblies. Since AP-2 is the best studied of the clathrin adaptors, we also understand those endocytic signals best that directly bind to AP-2 itself. Among those signals are short linear signals, in particular the tyrosine-based motifs that bind μ2 as well as the di-leucine motifs that bind to the interface of σ2 and α-subunits of AP-2 (Bonifacino and Traub, 2003; Traub and Bonifacino, 2013). Other endocytic signals do not bind directly to AP-2, but require accessory proteins or “co-adaptors”. For instance, the “NPXY” motif ([YF]XNPX[YF]) binds to the PTB domain [“PTB” stands for “phospho-tyrosine binding” but PTB domains can bind other motifs as well] of a number of EAPs, including ARH, numb, and Dab2. These additional accessory proteins are often referred to as “CLASPs” (clathrin-associated sorting proteins), “EAPs”, or “co-adaptors”. [Note to reader: There is also a class of microtubule-associated proteins named CLASP (cytoplasmic linker associated protein) which are not clathrin-associated proteins. To avoid confusion, we will use the terms “EAP” or “co-adaptor” in this review]. EAPs can be subdivided into several categories depending on their binding interactions. A full adaptor has four direct interactions: with lipids, with cargo, with clathrin, and with additional accessory endocytic proteins (Reider and Wendland, 2011). The AP-2 complex itself falls into this category. Other EAPs/co-adaptors contain only a subset of these binding sites and require additional binding partners to supply the rest of the interactions. For example, Eps15 has been shown to bind ubiquitinated cargos (via its UIM domains) and accessory proteins (via its EH domain), but to date direct binding to lipids or clathrin is not known (Reider and Wendland, 2011).

### A Large Arsenal of EAPs Enables Regulated Cargo- and Cell Type-Specific Endocytosis

What might be some of the physiological consequences of having multiple “full” adaptors and/or various “partial” co-adaptors expressed in the same cell? In fact, up to 60 different proteins can participate in the various steps of clathrin-mediated endocytosis (Merrifield and Kaksonen, 2014). Deletion of many of the EAPs does not completely disrupt clathrin-mediated endocytosis. In fact, clathrin-mediated endocytosis is notoriously robust, and continues or compensates rapidly for the loss of a single component. This likely represents the cooperative nature of building a large multimeric assembly from many components that are partially redundant (McMahon, 1999). Surprisingly, under some circumstances even AP-2 itself is dispensable. A large number of EAPs thus endows cells with endocytic robustness. This robustness creates experimental challenges in terms of testing the involvement of specific EAPs in the endocytosis of a cargo of interest (see McMahon and Boucrot, 2011) for a good discussion). In fact, global interference approaches might not be optimally suited to uncover the detailed molecular roles of individual EAPs. New technological advances have given rise to approaches that allow the analysis of the formation of single coated pits and their maturation in relation to the recruitment levels of individual EAPs. These approaches have shown that the kinetics of CCP formation and the concerted maturation to a productive coated pit are regulated by a multi-step cascade. In addition, compensatory mechanisms can be detected, allowing the analysis of phenotypes even when bulk transferrin uptake is not significantly impaired. This has led to a new framework of a concerted molecular checkpoint cascade that regulates CCP maturation. These new approaches with better signal-to-noise detection demonstrate that EAPs binding to the ear domain of α-adaptin are required for the concerted and regulated maturation of CCPs (Aguet et al., 2013).

Another consequence of having cargo-specific EAPs is that it allows cells to endocytose cargos with greatly differing cell surface abundance without competing for potentially limiting cargo binding sites on AP-2 itself (McMahon, 1999; McMahon and Boucrot, 2011). Increasing the abundance of cargo-specific EAPs, thus, leads to increased endocytosis of its specific cognate receptor cargos (containing the specific binding motif) without affecting overall endocytic levels of non-cognate cargos (not containing the specific binding motif).

Another physiological consequence of the large number of EAPs it that many are expressed in a cell-type restricted fashion, resulting in a versatile arsenal for regulating cargo selectivity. For some of them, multiple isoforms exist that can further expand the repertoire of combinatorial players. For the tetrameric AP adaptors themselves, several subunits have multiple isoforms. Some of these are expressed in a cell-type restricted fashion, including in neurons. For example, both the ubiquitously expressed AP-3A (subunit composition δ–β3A–μ3A–σ3) and the neuron-specific AP-3B (subunit composition δ–β3B–μ3B–σ3) complexes are expressed in the brain. AP-3 has been implicated in sorting synaptic vesicle membrane proteins to synaptic terminals (Danglot and Galli, 2007). Loss of the neuronal-specific AP-3B in μ3B mutant mice results in spontaneous epileptic seizure and impairment of GABA release due to a reduction in vesicular GABA transporters (Nakatsu et al., 2004). In humans, Hermansky–Pudlak syndrome (HPS) is associated with mutations in the gene encoding AP3-β3A and is characterized by albinism and defects in lysosome-related organelles (Starcevic et al., 2002). The lack of neurological defects reported in human HPS is most likely due to the presence of functional AP-3B (using the β3B subunit) in neurons. It thus appears that expression of cell type-restricted EAPs provides tissues with a versatile arsenal from which to choose in order to achieve exquisitely precise control over which receptor is endocytosed when and where. Our own attention was directed towards exploring the roles that the “EAP arsenal” might play in neuronal development after we made some surprising observations, first with regard to the neuronal- and cargo-specific roles that the ubiquitous EHD proteins play (Yap et al., 2010), and second with regard to the previously unknown role that doublecortin (DCX) plays in the regulated endocytosis of neurofascin (Yap et al., 2012).

Many genes have been identified over the past decades for their roles in various aspects of neural development. Many of them were first identified in *Drosophila* and *C. elegans*, but mammalian homologs exist and play crucial roles. The inventory list of genes involved in any particular developmental process is ever growing and sheds light on the genetic networks that regulate development. In addition, creating this inventory is a crucial steppingstone to proceed towards a molecular and mechanistic understanding of the identified gene products and their function in a complex network of proteins. Identifying the exact molecular role of each protein in the genetic network is an arduous and slow process, but exciting new discoveries are continuously being reported. The need to regulate endocytosis for proper neural development is highlighted by the fact that “famous” neurodevelopmental genes, such as numb, are now known to be EAPs. We will first discuss the functions of two EAPs with known neurodevelopmental roles, numb and disabled (Dab), and subsequently explore what is currently known about some other EAPs in regulating neuronal endocytosis for a specific subset of cargos.

## Numb—A Monomeric EAP Regulating Neural Development

**Figure 1:**
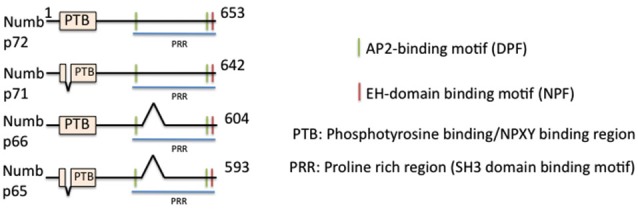
**Domain structure of numb and its isoforms**. The domain structure of full length numb (p72) and three splice isoforms is depicted. Binding regions are indicated by color bars.

In vertebrates, there are two genes encoding for the related Numb and numb-like (numbl) proteins (Zhong et al., 1996, 1997; Salcini et al., 1997). Numb is a peripheral membrane-associated protein with an amino-terminal phosphotyrosine-binding domain (PTB) and a C-terminal proline rich region (PRR), as well as EH-domain-binding motifs (two DPF and one NPF motif) (Figure 1). Numb binds its cargos via its PTB domain, binds the α-adaptin subunit of AP-2 via the C-terminal DPF motif, and binds other accessory proteins, such as Eps15 and EHD1 and 4, via the NPF motif. Numb thus meets the criteria for being an endocytic EAP. There are four spliced isoforms of numb (Dho et al., 1999; Verdi et al., 1999) which differ in the length of the PTB (either including or lacking a 11 aa insert) and PRR domains (including or lacking). Numb-like shares high sequence homology with Numb (Figure 1): it contains a PTB domain, the EH-binding motifs but lacks the PRR region and has an additional specific poly-glutamine repeat which is absent in Numb (Zhong et al., 1997). This isoform diversity adds additional variety into the cellular toolbox. The PTB region and the EH-binding motifs are conserved in vertebrate and *Drosophila* numb, suggesting these two regions are functionally relevant and that the roles of numb may be similar across vertebrate and invertebrate species.

### What are the Roles for Numb in Neural Development?

Numb has multiple functions in the nervous system and has been implicated in a dazzling number of processes, including cell fate determination, proliferation, neurogenesis, cell migration, cell adhesion, and axon outgrowth (Gulino et al., 2010). In the mouse embryo, Numb is expressed in all layers of the cortical plate, as well as the progenitor cells of the VZ (Zhong et al., 1997). During cortical neurogenesis and cell division, numb is asymmetrically localized to the apical membrane of the dividing cells, and subsequently segregated to the apical daughter cells that remain progenitors. A numb-knockout mouse displays premature neuronal differentiation in the forebrain, implying numb functions in maintaining progenitor cell numbers (Verdi et al., 1999; Zhong et al., 2000; Shen et al., 2002). In other contexts, Numb appears to be involved in promoting neurogenesis and differentiation instead. In fact, whereas at earlier stages (E10) the daughter cell inheriting numb remains a progenitor, later on in corticogenesis (E13) the daughter inheriting numb becomes a neuron (Shen et al., 2002). Studies from different numb knockout mouse models have given rise to conflicting results pointing to the diverse functions and complex regulation of Numb. For instance, in a second numb knockout mouse model, no premature differentiation phenotype was observed in forebrain, while impaired differentiation was detected in hindbrain and in cerebellum (Zilian et al., 2001; Klein et al., 2004), further suggesting that numb might also play a role in neurogenesis.

Numb has also been reported to play a critical role in cerebellar granule cell polarization during migration (Zhou et al., 2011): Conditional ablation of numb/numbl using the transcription factor math1-cre system in cerebellar granule cell precursors (GCPs) impairs BDNF-induced GCP migration both *in vitro* and *in vivo*. How can numb play so many, sometimes contradictory roles? Since numb is an endocytic adaptor, its particular role depends on the cargos whose trafficking numb regulates in different cellular contexts. Among the known numb cargos are receptors with well established roles in regulating developmental processes: Notch1, βAPP, β1 integrin, and TrkB. numb binds to the conserved NPXY sorting motifs in the cytoplasmic tail of these cargos and mediates their endocytosis. What is the current evidence that the brain phenotypes of numb knockouts are due to impaired endocytosis of a numb cargo?

### Numb-Mediated Endocytosis of Receptors Controls Neural Development

#### Notch

Numb was originally identified as an antagonist of Notch. Notch signaling regulates numerous developmental decisions and patterning events from worms to human. Notch signaling promotes radial glial identity and controls cell fate specification during development of the neocortex (Gaiano et al., 2000). It is likely that endocytosis of Notch receptor *per se* is regulated by Numb because the PTB domain of Numb binds the ram23 and the PEST regions of the Notch cytoplasmic tail directly, and numb-mediated inhibition of Notch requires the AP-2 specific subunit α-adaptin (Guo et al., 1996; Berdnik et al., 2002). The endocytic removal of Notch via numb reduces Notch receptor levels on the surface and reduces Notch signaling. Reduced Notch signaling then affects cell fate.

Additional molecular roles of numb are becoming apparent as well. Numb was found to have additional interacting partners, such as Par3 and atypical protein kinase C (aPKC), protein complexes which function in polarized cell migration. Knockdown of mPar-3 reduces Notch signaling activity and causes premature depletion of progenitor cells from the VZ (Bultje et al., 2009). Conversely, depletion of Numb/numblike abolishes this effect and increases Notch signaling activity. Numb and Numblike are thus required for mPar-3 function in regulating Notch signaling and neocortical neurogenesis.

#### TrkB

Granule cell precursors in the cerebellum migrate towards a source of the neurotrophin BDNF via activation of the BDNF receptor TrkB at the leading process of the cell (Zhou et al., 2007). Neurotrophin signaling via Trk receptors is well established to require endocytosis into endosomes. These endosomes contain activated Trk receptors and recruit a variety of signaling components, including PI3K, Ras-MAPK, and PLC-γ pathway components. Other cascades, such as PKA via elevated cAMP, have also been implicated in BDNF signaling. High levels of signaling takes place on these so-called “signaling endosomes” (Cosker and Segal, 2014). For some forms of signaling (such as long-range survival signaling by NGF), the signaling endosomes travel long distances back to the cell soma where they continue to signal and regulate transcription. There are also more locally restricted signaling events downstream of Trk receptor activation close to the sites of initial endocytosis that regulate axon growth and polarized mobility of migratory neuronal precursors. Neurotrophin signaling thus is one of the best-documented examples of an endocytosis-dependent signaling event.

**Figure 2:**
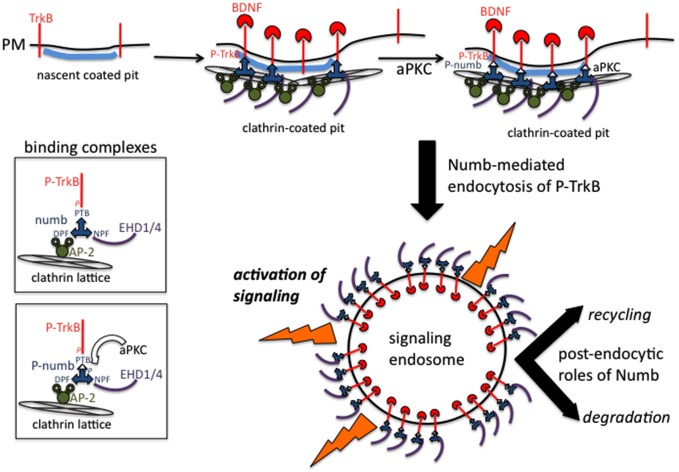
**Binding complexes of numb during clathrin-mediated endocytosis of TrkB receptors**. Activated TrkB binds to the PTB domain of numb which recruits AP-2, EHD proteins to the forming coated pit. Clathrin is recruited via AP-2 and leads to formation of a clathrin-coated endocytic vesicle. Numb also recruits other signaling effectors such as atypical protein kinase C (aPKC) and par3 (not depicted). Signaling downstream of activated TrkB commences at the plasma membrane and continues after endocytosis from signaling endosomes. Numb continues to play post-endocytic roles and might also regulate recycling vs. degradation trafficking.

**Figure 3:**
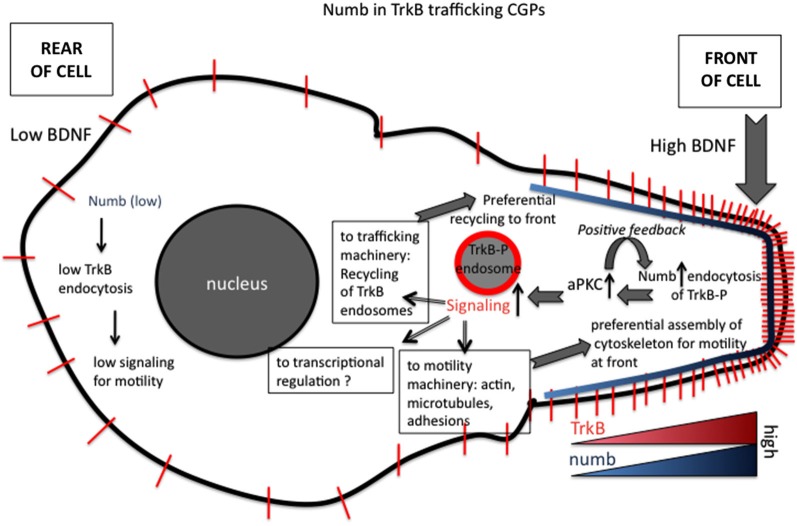
**Roles of numb in polarizing signaling responses downstream of BDNF in migrating granule cell precursors (GCPs) in cerebellar development**. Polarized distribution of numb at the leading process of a migrating GCP creates a positive feed-forward loop for directional migration. The following steps are proposed (based on Zhou et al., 2011). (1) Higher BDNF in front of migrating GCP. (2) Higher activation of TrkB. (3) Higher recruitment of numb. (4) Higher recruitment of downstream effectors, such as aPKC. (5) Higher numb leads to higher TrkB-P endocytosis via clathrin/AP-2. (6) Higher TrkB endocytosis leads to more TrkB-P in endosomes. (7) More TrkB-P in endosomes recruits more signaling effectors to endosomes. (8) Higher activation of Tiam and Rac to promote more actin assembly in front of cell. (9) Higher signaling promotes preferential recycling of TrkB back to surface at front. (10) TrkB distribution becomes more polarized near front.

How is endocytosis of activated TrkB and the subsequent signaling from signaling endosomes regulated in time and space? Numb is an endocytic co-adaptor for activated TrkB (Figure 2): Numb colocalizes with α-adaptin and TrkB in the leading processes, it interacts with the NPXY motif on TrkB after activation (via the numb PTB domain), and it promotes TrkB endocytosis and polarized localization to the leading process of migrating GCPs. Depletion of Numb either by shRNA or conditional knockout impairs BDNF-induced TrkB endocytosis and polarized localization of TrkB to leading processes of migrating GCPs. The polarized localization of numb leads to enhanced endocytosis of activated TrkB at the leading process, increased generation and accumulation of signaling endosomes locally in the leading process of GCPs, and consequently increased activation of downstream signaling cascades that promote local membrane trafficking (such as increased BDNF exocytosis) and cytoskeletal rearrangements that drive directional protrusions and mobility (Zhou et al., 2011; see Figure 3).

In addition to promoting local endocytosis of activated TrkB, numb also serves as an adaptor to recruit signaling components, such as aPKC, a well known regulator of polarized cellular events upstream of cytoskeletal rearrangements. Genetic deletion of numb/numbl impairs BDNF-induced aPKC activation in GCPs, suggesting BDNF stimulation of TrkB recruits Numb, which in turn acts as an adaptor to recruit and activate aPKC via the PTB region. In BDNF knockout mice, polarization of Numb at the front of GCPs and its interaction with aPKC are significantly decreased, suggesting BDNF regulates the polarization of Numb and its interaction with aPKC in migrating GCPs. Interestingly, Numb is also itself a target of aPKC. BDNF-induced phosphorylation of numb by aPKC increases its binding to TrkB and promotes the chemotactic response to BDNF. Taken together, Numb acts as an adaptor linking BDNF, an extracellular cue, to intrinsic cellular polarity machinery, including aPKC, via TrkB endocytosis and serves as a feed-forward loop to promote BDNF-induced directed GCP migration (Zhou et al., 2011; see Figure 3).

#### L1

Numb is also expressed in postmitotic neurons and functions in axonal outgrowth. Numb accumulates at the tip of growing axons in cultured hippocampal neurons and mediates endocytosis of L1/NgCAM for axon growth (Nishimura et al., 2003). Internalization of L1/NgCAM occurs preferentially in the central domain of migrating growth cones, followed by anterograde transport of L1/NgCAM-containing vesicles and subsequent recycling near the leading edge. Numb has been reported to co-localize with L1/NgCAM at the central region of growth cones, suggesting numb plays a role in regulating local L1/NgCAM internalization and maybe recycling for growth cone advance (Nishimura et al., 2003). Numb co-immunoprecipitates in a complex with L1 and AP-2 and is important for efficient endocytosis of L1/NgCAM. The PTB domain of numb is required for L1/NgCAM internalization, but L1/NgCAM does not itself contain an NPXY motif. It is currently not known if numb binds directly to L1/NgCAM and if so, via what binding motif. The regulation of L1/NgCAM endocytosis on numb is surprising since L1/NgCAM has its own tyrosine-based motif that binds directly to μ2 of the AP-2 tetrameric adaptor complex (Kamiguchi et al., 1998). It will be interesting to determine what aspects of L1/NgCAM function and signaling might be additionally influenced by numb.

### How is the Polarized Distribution of Numb Regulated?

In many cell types, numb localization is highly polarized to one side of the cell. This polarized distribution of numb is ideally suited to promote non-uniform endocytosis of numb cargos (see also Section TrkB). How is the polarized distribution of numb regulated? Some evidence points to important roles of the numb interacting polarity proteins par3 and aPKC. For example, numb mediates the endocytosis of integrins via binding of the PTB domain of numb to the NPXY motif in the cytoplasmic tail of integrins (Calderwood et al., 2003; Nishimura and Kaibuchi, 2007). In migrating ECV304 and HeLa cells, Numb polarizes toward the leading edge, accumulates around focal adhesions, and localizes to clathrin-coated structures at the substratum-facing surface of the leading edge. Its localization to clathrin-coated structures is α-adaptin dependent. Depletion of Numb by RNAi impairs both integrin endocytosis and cell migration. Par3-dependent phosphorylation by aPKC regulates the polarized localization of numb and its association with clathrin coated structures. Phosphorylated numb is released from the clathrin-coated structures and no longer binds integrin, suggesting aPKC negatively regulated functions of numb with respect to integrins. However, aPKC is required for polarized subcellular localization of Numb as knockdown of aPKC mislocalizes Numb to both the apical and basal surface leading to less enrichment in leading processes of the cells. Taken together, these results suggested that the polarized numb phosphorylation regulated by aPKC/Par3 complex is important for integrin endocytosis and integrin-substrate based cell migration (Nishimura and Kaibuchi, 2007). This is in contrast to the effects of aPKC phosphorylation on numb-TrkB binding which is increased by phosphorylation of numb (see Section TrkB).

Similarly in dividing sensory organ precursor (SOP) cells in Drosophila, a numb mutant with five PKC phosphorylation–deficient sites is mislocalized to both anterior and posterior sides of the cell. The results suggested that aPKC-mediated phosphorylation of numb regulates the asymmetric localization of numb in dividing cells (Smith et al., 2007), and that overall Numb functions relies on its phosphorylation status which determines its subcellular localization. Similarly, hyper-phosphorylated Numb after treatment with the phosphatase inhibitor calyculin-A dissociates from the AP-2 and Eps15 complex, indicating that phosphorylation at certain sites of numb negatively regulates its endocytic functions (Krieger et al., 2013). Interestingly, phosphorylation of Numb by Ca2+/calmodulin-dependent protein kinase I at positions S264 and S283 prevents Numb binding to AP-2, but promotes its interaction with 14-3-3 proteins (Tokumitsu et al., 2006), pointing to the participation of numb phospho-isoforms in distinct molecular complexes with presumably distinct functions in the cell. In addition, the recruitment of numb to endosomes is also regulated: phosphorylation of numb by AAK1 at position T102 redistributed numb from the plasma membrane to perinuclear endosomes (Sorensen and Conner, 2008).

There is thus strong evidence that numb participates in endocytosis and subsequent endosomal trafficking of NPXY-motif containing cargos during multiple processes in neural development, such as maintaining proper neurogenesis via regulated Notch trafficking, cerebellar migration via regulated TrkB trafficking, and axon growth via regulated L1 endocytosis. Since numb is localized in a polarized manner to only a part of the cell, endocytosis would be preferentially occurring on one side leading to polarized surface distribution of the numb cargo. This leads to changed signaling (for Notch or TrkB) or reduced adhesion (for integrin) or both, and changed cellular behavior. Since most of the numb cargos are signaling receptors that continue to signal after internalization, numb-mediated endocytosis of such a receptor would not stop signaling immediately, but allow signaling from endosomes. Furthermore, numb itself provides a scaffold for signaling components and thus participates directly in determining signaling output from its cargos. Subsequently, these receptors can be recycled to the surface for another round of ligand-binding and signaling (i.e., continued signaling) or be trafficked for degradation (i.e., termination of signaling).

### Multiple Splice Isoforms with Different Domain Structure Play Different Roles

In mammalian cells, numb isoforms are expressed in cell type-specific manner and localized differentially subcellularly dependent on the insert in the PTB domain. For instance, the absence of the 11 aa in the PTB domain results in more cytosolic and less membrane-associated Numb (Dho et al., 1999). The Numb isoform with long PRR domain is expressed transiently during early brain development and disappears prior to cell differentiation in P19 embryonic carcinoma cells, suggesting the isoform may function in promoting proliferation of progenitor cells. Conversely, the isoform with short PRR domain is expressed throughout neurogenesis in the developing brain and in adult brain, and during the course of retinoic acid-induced P19 cell differentiation (Dho et al., 1999; Verdi et al., 1999; Toriya et al., 2006). Expression of the short PRR Numb isoform in the outer optic anlage of the *Drosophila* larvae brain also promotes neuronal differentiation and reduces the levels of nuclear-localized Notch (Toriya et al., 2006), implying this short PRR isoform might mediate neuronal differentiation by regulating the endocytic sorting of Notch. Thus, different Numb isoforms might function in different developmental stages dependent on the time of expression and their respective subcellular localization.

## Other Co-Adaptor Proteins for NPXY-Motifs Involved in Signaling During Brain Development: Disabled Proteins

The NPXY motif is not only recognized by numb, but also by another set of co-adaptors, the Disabled (Dab) family of proteins. In addition, NPXY motifs bind to other adaptors, such as sorting nexins (SNX), Fe65, and X11/Mint (Uhlik et al., 2005; Stolt and Bock, 2006). Is there specificity of endocytic adaptors to a subset of NPXY-containing cargos, or do multiple endocytic adaptors play roles (either redundant or distinct) in trafficking the same cargos? Participation of a particular adaptor in cargo sorting would largely depend on cell-type expression, and on subcellular localization of the cargo.

### The Discovery of Disabled (Dab)

**Figure 4:**
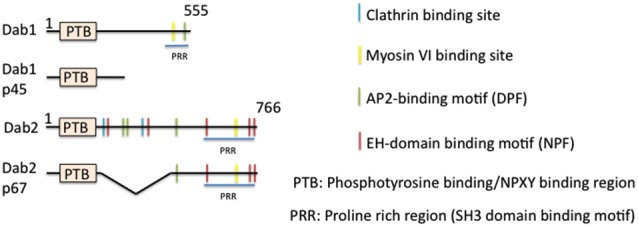
**Domain structure of mammalian disabled (Dab) and its isoforms**. The domain structure of disabled and of its isoforms is depicted. Binding regions are indicated by color bars.

One of the best studied “full” EAPs (i.e., binding sites for cargo, lipid, AP-2, and clathrin) is disabled (Dab), in particular the mammalian isoform Dab2 (Figure 4). Dab was first isolated from *Drosophila* in screens for mutations in genes that enhanced phenotypes of Abl−/− (Abelson tyrosine kinase, a non receptor tyrosine kinase) (Gertler et al., 1989, 1993). Although a later study showed that Dab is not the* bona fide* enhancer of Abl (Liebl et al., 2003), Dab is still a positive regulator of the Abl signaling pathway. Null mutations of Dab in *Drosophila* causes defects in motor axon patterning and epithelial morphogenesis, phenotypes resembling those of Abl mutants. Genetically, Dab acts in conjunction with Abl for proper growth and guidance of motor axons (Song et al., 2010). While it is not well established which cargos might undergo Dab-mediated endocytosis during axon guidance, a clear role for a clathrin-associated role for *Drosophila* Dab was shown by Kawasaki et al. in mature neurons: a novel Dab mutant that has a nonsense mutation within the conserved N-terminal PTB domain exhibits impaired synaptic function. The experiments indicated that Dab is involved in clathrin-mediated endocytosis for rapid clearance of neurotransmitter release sites, which is important for subsequent vesicle priming and refilling of the readily releasable pool (Kawasaki et al., 2011).

In mammals, the situation is more complicated. There are two members of the Dab family identified thus far, Dab1 and Dab2. Dab is most closely homologous to Dab2. Dab1 was originally isolated as a Src-binding protein (Howell et al., 1997a), whereas Dab2 was identified as a phosphoprotein regulated by colony-stimulating factor CSF1 (Xu et al., 1995). Dab1 is highly neuron-enriched and functions in cell positioning during brain development, whereas Dab2 regulates endodermal cell organization during embryogenesis (Howell et al., 1997b, 1999a; Morris et al., 2002; Yang et al., 2002) and is widely expressed. Dab1 and Dab2 are multi-domain cytoplasmic adaptor proteins and function in mediating several signaling pathways. Structurally, Dab1 contains a PTB-domain, clathrin adaptor AP-2-, SH3 domain-, and myosin-binding sites at its unique C-terminus (Figure 4). Similarly to Dab1, Dab2 is composed of an N-terminal PTB domain which is 63% identical to that of Dab1. The Dab2 C-terminus contains clathrin- and EH-domain-binding sites, which are absent in Dab1, in addition to AP-2-, SH3-domain-, and myosin VI-binding sites, which are shared with Dab1. The Dab N-terminal PTB domain is known to bind preferably to non-phosphorylated NPXY internalization motifs present in the cytoplasmic tails of βAPP and LDL-related family proteins, such as LDLR/VLDLR, LRP, ApoER2, and megalin (Trommsdorff et al., 1998, 1999; Howell et al., 1999b; Oleinikov et al., 2000). Additionally, the PTB domain is required for recruitment to the plasma membrane by binding to phospholipids.

### Dab2 as an Endocytic Adaptor

Most studies of Dab2 highlight its roles in clathrin-mediated endocytosis. For instance, loss of Dab2 results in decreased endocytosis of transferrin within visceral endoderm and fewer early endosomes in conditional Dab2 knockout mice. Other tissues, such as the kidney, are also affected. In NIH3T3 cells, Dab2 localizes very close to the plasma membrane at CCPs that are also positive for AP-2, but is absent from early endosomal and lysosomal compartments. Its localization to clathrin-coated vesicles is dependent on the clathrin- and AP2-binding sites located in the C-terminus as the Dab2 p67 isoform lacking the binding sites (Figure 4) do not associate with CCPs and is defective for receptor endocytosis (Morris and Cooper, 2001; Mishra et al., 2002; Maurer and Cooper, 2006). Mishra at al. demonstrated that Dab2 interacts directly with clathrin independently of AP-2 association, and engages soluble clathrin trimers via its multiple clathrin binding sites to assemble complete polyhedral clathrin cages. Importantly, clathrin recruitment by Dab2 does not affect its interaction with AP-2 (Mishra et al., 2002). Many lines of evidence thus demonstrate that Dab2 is a* bona fide* endocytic co-adaptor in clathrin-mediated endocytosis of NPXY-containing cargos, such as members of the LDL receptor family.

### Disabled in the Mammalian Nervous System: Dab1 Regulates Neural Development

There is great interest in Dab1 among developmental neuroscientists since it is highly expressed in neurons, and it has been linked to signaling of major developmental receptors pathways, including reelin (via the reelin receptors ApoER2 and VLDLR), and βAPP. Two mutant mice arising from spontaneous mutations in Dab1, Scrambler and yotari (Sweet et al., 1996; Yoneshima et al., 1997), display inverted cortical lamination, abnormal positioning of neurons, and aberrant orientation of cell bodies and fibers, similar to the reeler mouse which lacks the ligand reelin itself (Falconer, 1951; Goffinet, 1979; Sheldon et al., 1997; Ware et al., 1997). A targeted disruption of the PTB domain of Dab1 resulted in a Dab1 null with no Dab1 mRNA detectable. This Dab1 null mouse also exhibits identical phenotypes to the reeler mouse (Howell et al., 1997b). The Dab1 null mouse is ataxic and dies prematurely between P20 to P30. No clear lamination into layers could be distinguished in either the cerebral cortex or hippocampus. Similarly, the development of the cerebellum is severely affected, unfoliated and small in size. The mutant Purkinje cells are present in the central mass with their dendrites oriented randomly. This suggested that interaction of Dab1 with reelin receptors is essential for reelin-dab1-mediated neuronal migration. Since the phenotypes of the Dab1 null mouse resembles those of the reeler mouse, Dab1 and reelin are part of a single genetic signaling pathway (Rice and Curran, 2001).

### The Mechanism of Dab1 Regulation of Reelin Signaling

The PTB domain of Dab1 directly interacts with the NPXY motifs located in the cytoplasmic tails of the reelin receptors VLDLR and ApoER2 (Trommsdorff et al., 1999). Reelin-signaling is initiated by direct binding of reelin to the extracellular domains of VLDLR and ApoER2 (D’Arcangelo et al., 1999; Hiesberger et al., 1999; Rice and Curran, 2001) and subsequently leads to increased levels of phosphorylated Dab1. Reelin-induced phosphorylation of Dab1 leads Src family of non-receptor tyrosine kinases and recruitment of Crk family adaptor proteins (Park and Curran, 2008) which trigger downstream intracellular signaling cascades, such as activation of AKT, PI3K and Erk1/2. Tyrosine phosphorylation of Dab1 at baseline level prior to reelin stimulation is essential for the subsequent reelin-induced activation of Src (Arnaud et al., 2003; Bock and Herz, 2003). VLDLR and ApoER2 are two essential components of the Reelin-signaling pathway. The VLDLR–knockout mouse has smaller cerebellum, whereas the ApoER2-deficient mouse displays two layers of CA1 region. Importantly, the VLDLR and ApoER2 double knockout mouse exhibits identical phenotypes to that observed in reeler/scrambler mice (Trommsdorff et al., 1999). Howell et al. (2000) identified several tyrosine phosphorylation sites clustered close to the PTB domain of Dab1 that are important for cell positioning during brain development. They demonstrated that a mutant mouse with all the identified potential phosphorylation sites mutated display phenotypes identical to those observed in reeler/Dab1-null mice (Howell et al., 2000).

Surprisingly, despite the fact that Dab1 binds the NPXY motif and contains an AP-2 binding site, evidence for a direct role in endocytosis is lacking, and no detailed study demonstrating its association with clathrin-AP2 complex in the regulation of reelin/ApoER2/VLDLR endocytosis exists. Similarly, the endocytic trafficking routes of reelin/receptor-bound Dab1 signaling complex remain poorly understood. Whether phosphorylation affects the function of Dab1 in the endocytic trafficking of reelin-ApoER2 complex, such as dissociation of phosphorylated Dab1 from the complex, remains to be answered. So far there are only a handful of papers showing Dab1-signaling complex in endosomal-like structures. Brian Howell was the first to show Dab1 diffusely in the cell soma and enriched in the axon where it co-localizes with βAPP in small vesicular-like structures (Howell et al., 1999b). Subsequently, Curran’s group showed Dab1 co-localization with APLP1 in membrane ruffles and vesicular structures (Homayouni et al., 2001). Although Hiesberger and D’Arcangelo both reported that binding of reelin to VLDLR/ApoER2 on the cell surface mediates its internalization into vesicles (D’Arcangelo et al., 1999; Hiesberger et al., 1999), it is not clear whether vesicles that are positive for internalized reelin also contain Dab1. Similarly, Leeb et al. did not show the presence of Dab1 in EEA1-positive early endosomes colocalizing with clusterin ligand-bound ApoER2/VLDLR complexes (Leeb et al., 2014).

In 2002, it was reported that deletion of the C-terminal region of Dab1 (corresponding to the p45 isoform) (Figure 4) has no affect on cortical neuronal migration *in vivo* (Herrick and Cooper, 2002), and the isoform still appears to be phosphorylated, indicating that the AP-2 and SH-domain binding sites are not crucial for activation of reelin-Dab1 signaling during migration. This result argued strongly that Dab1 works in endocytosis-independent pathways during corticogenesis downstream of reelin signaling. However, a mutant mouse expressing only a single copy of the p45-Dab1 isoform (Dab1^p45/−^, i.e., p45 hemizygote) displays disrupted neocortex and hippocampus development, where the marginal zone of the neocortex shows the presence of late-born neurons, while the CA1 region of hippocampus is separated into two layers. The same paper showed that a mouse carrying one copy of full length p80-Dab1 (Dab1^p80/−^, i.e., p80 hemizygote) shows no migration defect. This observation suggested that the C-terminus of Dab1 might have important functions required for signaling in specific neurons during later stages of brain development.

Is there evidence for or against a role of Dab1 in clathrin-mediated endocytosis? A report by Morimura et al. revealed that tyrosine phosphorylated Dab1 is recruited to the plasma membrane and co-localizes with reelin/receptor complexes in puncta on the cell periphery of cortical neurons of reeler mice after addition of reelin (Morimura et al., 2005). They observed that two minutes after reelin wash-out, phosphorylated Dab1 still co-localizes with reelin in puncta. However, the phosphorylated Dab1 appears to dissociate from the receptor complex 20 min after removal of unbound reelin, the time point when endocytosis is presumably completed, suggesting phosphorylated Dab1 does not traffic extensively with the endocytosed reelin/receptor complex after internalization. Importantly, inhibition of Dab1 phosphorylation by the src kinase inhibitor PP2 prevents internalization of reelin, leaving reelin co-localized with Dab1 near the plasma membrane for a prolonged period. The study concluded that Dab1 regulates cell surface expression of reelin receptors by promoting translocation of the receptors to the plasma membrane, and that phosphorylation of Dab1 initiates intracellular trafficking of reelin/receptor complex in neurons. It is not known whether the puncta positive for reelin and Dab1 at the two-minute time point are on the cell surface or whether they are endocytosed vesicles as the immunostaining for reelin and Dab1 was done after permeabilization. Since endocytosis can be extremely fast, the two-minute time point might include Dab1 on endosomes. This remains to be established more firmly.

In addition, Dab1 has been implicated in regulating the processing and trafficking of βAPP and ApoER2, but this might be during post-endocytic trafficking. A study by Rebeck’s group demonstrated that interaction with Dab1 increases the cell surface levels of ApoER2 and βAPP, increases cleavage and secretion of the extracellular domain of the proteins and decreases levels of βAPP C-terminal fragments (Hoe et al., 2006). The effects are NPXY motif- and PTB domain-dependent. Treatment with reelin increases the interaction between Dab1 and βAPP or ApoER2 and significantly lowers the levels of secreted Aβ (Hoe et al., 2006). Notably, Dab1 KO mice have higher levels of Aβ compared to littermate controls, implicating the Dab1-βAPP interaction in regulating Aβ production. Overall the data indicated that Dab1 is involved in regulating the intracellular trafficking and processing of βAPP and of ApoER2 facilitated by reelin. The question of whether Dab1 acts as a scaffold/stabilizer for βAPP/ApoER2 on the cell surface to prevent the proteins from being sorted into endosomal compartments that facilitate Aβ production, or as an adaptor to ensure correct endocytic sorting remains to be investigated.

In addition to direct binding of Dab1 to endocytic machinery, there might also be Dab1 interactions with additional co-adaptors that mediate endocytosis of Dab1 cargos. For example, Fuchigami et al. demonstrated that Dab1 co-localizes with ApoER2 and CIN85 in vesicle-like structures at the plasma membrane (Fuchigami et al., 2013). CIN85 is an adaptor protein involved in endocytosis of receptors including EGFR and dopamine receptor (Dikic, 2003; Shimokawa et al., 2010) by binding to the cbl-EGFR complex via its SH3 domains and to endophilins via its PRR domain. In addition, CIN85 interacts with the PRR region of Dab1. Treatment with a reelin fragment containing the ApoER2/VLDLR binding sites (called the “reelin-repeats”) in the presence of Dab1 induced CIN85 localization to early endosomes which contained EEA1, the reelin-repeat fragment and ApoER2 in neurons. Since CIN85 binds phosphorylated Dab1, it is highly possible that interaction of Dab1 with Cin85 might facilitate Dab1-mediated internalization of the receptor complex and possibly trafficking to early endosomal compartments.

## Endocytosis Regulation in Neurons—What Other Players are Known?

Numb plays crucial role in the nervous system but is expressed widely outside the nervous system and plays important roles in many places. Are there also neuronal-specific EAPs that might play neuronal-specific roles? And how are cargos with different endocytosis signals endocytosed? What other EAPs and co-adaptors have functions in nervous system development? There are likely many still undiscovered and their roles are largely unknown. We will discuss below the identification of one putative neuronal endocytic co-adaptor, namely doublecortin.

### Doublecortin (DCX): A Critical Regulator of Cell Migration and Axon Tract Formation

**Figure 5:**
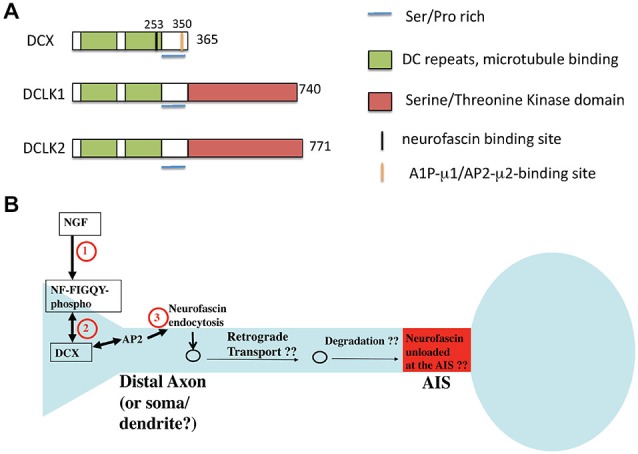
**Domain structure of DCX and related proteins DCLK1 and 2. (A)** The domain structure of DCX and DCLK1/2 is depicted. Binding regions are indicated by color bars. **(B)** Model of DCX as an endocytic adaptor (based on Yap et al., 2012). DCX binds to the phosphorylated FIGQY motif of the adhesion receptor neurofascin. DCX can bind to AP-2 via its C-terminal YLPL motif and mediate endocytosis of phospho-neurofascin. Neurofascin endocytosis reduces its levels on soma and dendrites and contributes to its gradual accumulation on the axon initial segment (AIS). If endocytosed neurofascin is subsequently degraded or recycled to the AIS is not known.

Doublecortin (DCX) was first identified as the major gene causing X-linked subcortical laminar heterotopia in female and lissencephaly syndrome in male patients (des Portes et al., 1998a,b). These defects manifest as cortical and hippocampal layering defects, leading to phenotypes including epilepsy and mental retardation. The layering defects are due to problems with proper neuronal migration. DCX has two closely related homologs, DCLK1 and DCLK2, both of which contain a kinase-domain at their C-termini, absent in DCX (Figure 5). DCX is prominently expressed in young postmitotic neurons, with transient expression detected during adult neurogenesis. Unlike DCX, DCLKs are expressed across the nervous system throughout adulthood. Although DCX is the causative gene for X-linked Lissencephaly in humans, DCX knockout mice display only mild neurodevelopmental delays in the cortex, and cortical lamination in the adult DCX KO mouse is indistinguishable from wild type. In contrast, severe lamination defects are observed in the CA3 region of the hippocampus (Corbo et al., 2002). However, DCX/DCLK1 double KO mice exhibit severely disrupted lamination in the cortical plate, suggesting compensation from DCLK1 might have contributed to the mild cortical phenotypes observed in the DCX KO mouse. Since DCX and DCLK1 are highly related in protein sequence (Figure 5), functional redundancy between the two proteins is very likely. In addition, cooperative functions of both proteins in axon outgrowth and in mediating fiber tract decussation have been reported (Deuel et al., 2006; Koizumi et al., 2006; Tanaka et al., 2006). Study on an allelic series of DCX/DCLK1 KO mice with cresyl violet staining reveals a dosage-dependent effect on cortical lamination. Disorganized cytoarchitetonics with dispersion of layer2/3 in the cortex was observed in DCX mutant mice with one copy of DCLK removed. Similar phenotypic defects were detected in DCLK1 mutant mice lacking a copy of DCX. In addition, Koizumi et al. (2006) observed a dosage-dependent interaction between DCX and DCLK1 in commissural fiber tract formation. Removal of one copy of DCLK1 in DCX-null mice causes hypoplastic corpus callosum and thin anterior commissures, whereas anterior commissures are hypoplastic in DCLK1 mutant mice lacking a copy of DCX. On the other hand, deletion of all four copies of DCX and DCLK1 results in disappearance of the corpus callosum, anterior commissures and hippocampus commissures.

### Microtubule-Based Roles for DCX

On the molecular level, DCX binds microtubules (MTs) via its two DC repeats to stabilize MTs and promote MT polymerization (Figure 5A). DCX has been reported to function with Lis1 and dynein to mediate nucleus-centrosome coupling in neuronal migration. Depletion of DCX in neurons results in delayed centrosomal-nuclear movement, whereas DCX overexpression is able to rescue the nucleus-centrosome coupling defect caused by dynein inhibition and increases the migration rates (Tanaka et al., 2004). These defects are largely attributed to the microtubule-binding activity of DCX. Similar to Dabs, DCX is a phosphoprotein and is known to be a substrate for kinases such as PKA, Cdk5, MAPK8 (aka JNK1), and others. Phosphorylation of DCX regulates its MT binding activity and its localization at the leading processes of migrating neurons: Cdk5-mediated phosphorylation of DCX at Ser297 increases DCX binding to MTs, leading to MT stabilization and promotion of tubulin polymerization, events crucial for neuronal migration (Tanaka et al., 2004).

In addition to microtubule binding, DCX also binds several other proteins (Caspi et al., 2000; Friocourt et al., 2001; Kizhatil et al., 2002; Tanaka et al., 2004; Liu et al., 2012). The roles of these binding interactions are still under investigation. Analysis of double knockout mice for DCX and DCLK1 have uncovered a previously unsuspected role for the DCX proteins in axon outgrowth and dendrite branching and demonstrated aberrant trafficking of synaptic vesicle proteins (Deuel et al., 2006). In neurons cultured from DCLK1 KO with additional DCX knockdown, VAMP2 and synaptophysin accumulate in the cell body, but are completely absent from axons, a drastic contrast from the WT where both proteins are detected in the cell body and the axons. In a follow-up study by Liu et al., the VAMP2 transport defect found in DCX/DCLK1-deficient neurons is caused by the mislocalization of Kif1a. Interestingly, a disease-associated DCX mutation impairs Kif1a motility and disrupts Kif1a-mediated VAMP2 transport from the soma to the neurites. The finding suggested that DCX/DCLK1 is involved in neuronal migration and axonal outgrowth via its interaction with Kif1a on the microtubules for transport regulation (Liu et al., 2012).

### DCX as an Endocytic Co-Adaptor for Cell Adhesion Molecules

Several of the other reported defects in DCX deficiencies have led to suggestions that DCX is involved in vesicle trafficking. Defects in synaptic vesicles and synaptic vesicle proteins (such as VAMP2) have all been found (Friocourt et al., 2003; Deuel et al., 2006). The exact molecular mechanisms of such putative trafficking effects of DCX, though, are not known, nor are the cargos. We recently found that DCX might act as an endocytic adaptor and modulate the surface distribution of the cell adhesion molecule neurofascin in developing cultured rat neurons (Yap et al., 2012). DCX binds directly to the cytoplasmic tail of neurofascin via a binding site dependent on residue G253 in DCX (Kizhatil et al., 2002). In contrast to numb and Dab2 which interact with the α subunit of AP-2, DCX associates with the AP2-μ2 subunit via a YLPL motif located in the DCX C-terminus (Friocourt et al., 2001; Figure 5A). The YLPL motif conforms to the YXXΦ class of tyrosine-based sorting motifs and is absent in DCLK1/2. Since DCX had been reported to bind to neurofascin (Kizhatil et al., 2002), we tested if DCX mediated the endocytosis of neurofascin. In fact, depletion of DCX diminishes endocytosis of endogenous neurofascin in cultured neurons (Yap et al., 2012). It was previously shown that a cytoplasmic motif in neurofascin (FIGQY) binds to ankyrin when unphosphorylated. In contrast, the phosphorylated FIGQY motif has low affinity for ankyrin, but high affinity for DCX (Kizhatil et al., 2002). Signaling of neurotrophins leads to phosphorylation of the FIGQY motif in the cytoplasmic tail of neurofascin, thereby increasing its affinity for DCX. We proposed (Yap et al., 2012) that local signaling via NGF leads to phosphorylation of the FIGQY motif in neurofascin, which can then bind to DCX locally. Neurofascin is then endocytosed in a DCX-dependent manner from the neuronal plasma membrane, especially in the soma and dendrites (Figure 5B). The endocytic machinery required for this event is not yet clear but may involve AP-2 clathrin adaptors. Since we observed the most robust endocytosis of neurofascin in young neurons, DCX-mediated modulation of neurofascin levels could play a role during development. Whether DCX interacts with and modulates sorting of other cargos via clathrin/AP-2 mediated endocytic trafficking remains to be investigated.

## Postendocytic Trafficking—Same Signals, Different Machinery?

Given the fact that the μ-subunits of tetrameric AP complexes recognize and interact with similar cytoplasmic motifs in cargos, it is believed that endocytic trafficking of a cargo involves several AP complexes to act in conjunction with each other in a sequential manner for sorting a cargo to its final destination. Yuzaki’s group showed that NMDA-induced AMPAR trafficking to the late endosome requires sequential interactions of stargazin, a transmembrane AMPA receptor regulatory protein (aka TARP-γ2), with AP-2 followed by AP-3 during NMDA-dependent long-term depression. Stargazin promotes association of AMPAR with AP-2 and AP-3, and mediates formation of ternary complexes containing AMPAR and the AP complexes. Inhibition of the stargazin interaction with AP-2 impairs NMDA-induced AMPAR endocytosis, whereas inhibition of the stargazin interaction with AP-3 disrupts late endosomal/lysosomal trafficking of AMPAR, thereby leading to recycling of AMPAR back to the cell surface (Matsuda et al., 2013). Similarly, Schachner’s group showed that NCAM promotes switching of synaptic vesicles recycling from an AP-3 to an AP-2–dependent mechanism during synapse maturation (Shetty et al., 2013). Both studies suggested that the sequential interaction of a cargo with different AP complexes for its sorting from one endosomal compartment to the subsequent compartment along the endocytic pathway is essential for proper functions in neurons.

### Post-Endocytic Roles of Numb

Numb localizes not only to the plasma membrane where it can aid endocytosis, but also to endocytic organelles as well as the TGN, and co-traffics to endosomes with endocytosed receptors (Santolini et al., 2000). In addition to its function in clathrin/AP2-mediated internalization, growing evidence thus implicates Numb in regulating endosomal sorting of receptors post-endocytosis. This is not surprising since many adaptor binding proteins bind more than one of the 5 tetrameric adaptors, such as AP-1 and AP-2. In fact, *Drosophila* numb physically interacts with the AP-1 complex by co-immunoprecipitation (Cotton et al., 2013).

#### Notch

Numb has been implicated in regulating the post-endocytic trafficking of Notch1. Endocytosis is required to maintain the steady state level of Notch receptors (Le Borgne et al., 2005). Mammalian Notch1 is known to constitutively internalize and traffic to recycling and late endosomal compartments. Despite the fact that numb-mediated inhibition of Notch signaling requires α-adaptin, it is still unknown whether Numb directly regulates the endocytosis or it regulates the endocytic trafficking of Notch after internalization via its association with Eps15 and α-adaptin (Le Borgne et al., 2005). What is known is that interaction of numb with the intracellular domain of Notch (Notch ICD) recruits the E3-ubiquitin ligase itch to the membrane-tethered Notch and leads to polyubiquitination and degradation of Notch ICD. In a study using mammalian cell lines, overexpression of Numb promotes trafficking and degradation of Notch 1, whereas depletion of numb facilitates recycling of Notch1. Numb mutants defective for binding to Itch, Eps15 or adaptin, fail to promote Notch 1 degradation, suggesting Numb suppresses Notch activity by regulating post-endocytic sorting pathways that lead to the degradation of Notch (McGill and McGlade, 2003; McGill et al., 2009).

#### Sanpodo

In *Drosophila*, interaction of Numb with α-adaptin is required for numb-mediated asymmetric cell division. During asymmetric cell division of SOPs, numb distributes asymmetrically between two daughter cells (called pIIa and pIIb), which is important for the subsequent binary cell fate decision. Hutterer et al reported that internalization of Sanpodo, a transmembrane protein required for Notch signaling in *Drosophila*, is mediated by α-adaptin via interaction with Numb (Hutterer and Knoblich, 2005). However, two recent studies demonstrated that Numb is not essential for the internalization of Sanpodo. The bulk of AP-2-dependent Sanpodo endocytosis still occurs in Numb mutant SOPs (Cotton et al., 2013; Couturier et al., 2013a,b). The studies proposed that Numb interacts with the sanpodo-Notch complex at early endosomes in concert with AP-1 to regulate the endosomal trafficking of the complex. The interaction of numb with sanpodo blocks the recycling of Notch back to the plasma membrane, leading to the asymmetric distribution of Notch along the pIIa-pIIb cell contact interface. A recent finding by Couturier et al. (2014) using dual GFP/cherry-tagged sensors in live SOP cells further confirmed that sanpodo is internalized into early/sorting endosomes in the Numb-inheriting daughter cell and sorted toward late endosomes, a process which is dependent on Numb. Similar to mammalian cells then, numb in flies mediates inhibition of Notch via its regulation of cargo sorting towards late endosomes and inhibition of recycling (Couturier et al., 2014). Some of the seemingly disparate roles of numb on Notch signaling might thus be due to differential post-endocytic sorting of numb cargos towards either recycling or degradative pathways. Depending on the cell type or developmental timing, numb-dependent Notch trafficking could either lead to increased signaling (via recycling) or decreased signaling (via degradation).

#### Cadherins

Cadherins are required for adhesion and polarity of radial glia cells (RGCs) in the cortex as depletion of cadherins by shRNA results in loss of end-feet and of bipolar morphology. Numb and Numbl are required for the maintenance of cadherin-mediated radial glial adherens junctions (Rasin et al., 2007). EM analysis demonstrated that Numb was enriched just basally of the apical end-feet of interphase RGCs and localized to internalized cadherin-containing Rab11-positive endosomes. Numb physically interacts with the cadherin/catenin complex via its PTB and C-terminal domains, and proper localization of cadherins requires Numb/numbl. Inactivation of Numb and Numbl in conditional double knockout mice decreases basolateral insertion of cadherins and mislocalizes cadherins to the cytoplasm and to apical membrane regions of RGCs. The changed cadherin distribution is likely the reason for the disrupted adherens junctions and loss of polarity observed in the mice. This results in progenitor cells dispersion and disorganized cortical lamination. In contrast, overexpression of Numb and numbl prolongs cadherin-dependent RGC apical attachment and polarization. Thus, Numb plays a critical role in ensuring correct trafficking of cadherins, a process required for the maintenance of adherens junctions during neurogenesis (Rasin et al., 2007).

### Sorting Nexin 17 (SNX17): Regulating Post-Endocytic Trafficking of ApoER2

In addition to binding to Dab1, ApoER2 has recently been reported to interact with SNX17 via its cytoplasmic NPXY motif for its endocytic trafficking and receptor signaling (Sotelo et al., 2014). SNX17 is a cytosolic protein that is highly expressed in mouse brain and localized to early endosomes. SNX17 is involved in the endocytic trafficking of membrane proteins, including LRP1, integrins, and P-selectin. For instance, SNX17 has been reported to mediate recycling of integrins. SNX17 binds integrin and prevents degradation in lysosomes (Böttcher et al., 2012; Steinberg et al., 2012). Similarly, SNX17 co-localizes with endocytosed ApoER2 and facilitates the trafficking from early endosomes to recycling endosomes (Sotelo et al., 2014). Depletion of SNX17 causes retention of ApoER2 in Rab5-positive early endosome compartments, leading to a decrease of ApoER2 in Rab11 recycling endosomes. The defect in ApoER2 recycling causes low surface level of ApoER2 and leads to dendritic outgrowth defects. Interestingly, accumulation of ApoER2 in the early endosome of SNX17-deficient neurons promotes proteolytic cleavage of its C-terminal domain. Furthermore, downregulation of SNX17 also promotes reelin-induced degradation of ApoER2, and dampens the activation of downstream reelin effectors, such as the phosphorylation of Dab1. The data suggested that SNX17 is an endosomal adaptor that regulates the post-endocytic trafficking and recycling of ApoER2. It is via this post-endocytic role that SNX17 participates in reelin-receptor signaling (Sotelo et al., 2014). Therefore, in neurons some co-adaptors such as Numb (and possibly Dab1) regulate endocytosis of NPXY-containing cargos at the plasma membrane as well as their postendocytic sorting. Other co-adaptors, such as SNX and likely other still unidentified co-adaptors, might be additionally involved in the subsequent steps of sorting following endocytosis.

## Conclusions

In this review, we laid out some of the emerging themes of how regulating the endocytosis and endosomal trafficking of critical receptors contributes to orchestrating developmental decisions in the nervous system. We highlighted some of the better understood endocytic co-adaptors, but many other pathways are similarly coordinated by endocytic regulation. For instance, Wnt signaling via disshevelled is dependent on endocytosis (Onishi et al., 2013). In particular, we discussed examples highlighting the following concepts:

(1) Monomeric EAPs enable interactions of their cognate cargos with the AP-2/clathrin machinery via binding sites that are distinct from endocytic signal binding sites on AP-2 itself. They do thus not compete with cargos using different signals or distinct EAPs. Changing the expression levels of one EAP can therefore change the endocytic rate of just the cognate cargos without affecting other non-cognate cargos.
(2) NPXY-containing cargos can interact with more than one monomeric co-adaptor. The consequences of which co-adaptors binds to which cargo include differential recruitment of different effector cascades, decision to recycle or degrade, effects on residence time in endosomes which affects signaling strength.
(3) EAPs do more than promote endocytosis of their cognate cargos. They can participate in multiple distinct complexes while bound to their cargos and thus regulate signaling. They are regulated scaffolds that can recruit signaling effectors.
(4) Distinct isoforms of EAPs have distinct functions and can affect the ultimate signaling output from the cognate ligand-receptor system.
(5) Many monomeric co-adaptors act at multiple steps along the endocytic-endosomal pathways and sequentially engage distinct set of tetrameric AP complexes as well as other machinery.

Given the large number of receptor pathways, we look forward to many more exciting discoveries of the diverse roles of EAPs in neural development.

## Conflict of Interest Statement

The authors declare that the research was conducted in the absence of any commercial or financial relationships that could be construed as a potential conflict of interest.
